# Elderberry Leaves with Antioxidant and Anti-Inflammatory Properties as a Valuable Plant Material for Wound Healing

**DOI:** 10.3390/ph17050618

**Published:** 2024-05-10

**Authors:** Elżbieta Studzińska-Sroka, Magdalena Paczkowska-Walendowska, Zuzanna Woźna, Tomasz Plech, Piotr Szulc, Judyta Cielecka-Piontek

**Affiliations:** 1 Department of Pharmacognosy and Biomaterials, Poznan University of Medical Sciences, Rokietnicka 3, 60-806 Poznan, Poland; elastudzinska@ump.edu.pl (E.S.-S.); zuzka.wozna@gmail.com (Z.W.); jpiontek@ump.edu.pl (J.C.-P.); 2 Department of Pharmacology, Medical University of Lublin, Radziwillowska 11, 20-080 Lublin, Poland; tomasz.plech@umlub.pl; 3 Faculty of Medicine, Mazovian Academy in Płock, 09-402 Płock, Poland; 4 Department of Agronomy, Poznań University of Life Sciences, Dojazd 11, 60-632 Poznan, Poland; piotr.szulc@up.poznan.pl

**Keywords:** *Sambucus nigra*, polyphenolic compounds, biological activity, antioxidant, anti-inflammatory, wound healing, PCA

## Abstract

*Sambuci folium* (elderberry leaves) have been used in traditional medicine, mainly externally, to treat skin diseases and wounds. Therefore, the aim of this study was to screen the biological activity of elderberry leaves (antioxidant potential and possibility of inhibition of tyrosinase and hyaluronidase enzymes) combined with phytochemical analysis. For this purpose, a phytochemical analysis was carried out. Elderberry leaves of 12 varieties (“Sampo”, “Obelisk”, “Dwubarwny”, “Haschberg”, “Haschberg 1”, “Koralowy”, “Sambo”, “Black Beauty”, “Black Tower”, “Golden hybrid”, “Samyl”, “Samyl 1”) in two growth stages. The compounds from the selected groups, phenolic acids (chlorogenic acid) and flavonols (quercetin), were chromatographically determined in hydroalcoholic leaf extracts. All tested elderberry leaf extracts showed antioxidant effects, but the most promising potential: very high compounds content (TPC = 61.85 mg GAE/g), antioxidant (e.g., DPPH IC_50_ = 1.88 mg/mL; CUPRAC IC_0.5_ = 0.63 mg/mL) and optimal anti-inflammatory (inhibition of hyaluronidase activity 41.28%) activities were indicated for older leaves of the “Sampo” variety. Additionally, the extract obtained from “Sampo” and “Golden hybrid” variety facilitated the treatment of wounds in the scratch test. In summary, the best multidirectional pro-health effect in treating skin inflammation was specified for “Sampo” leaves II extract (leaves during the flowering period); however, wound treatment was noted as rich in chlorogenic acid younger leaf extracts of the “Golden hybrid” variety.

## 1. Introduction

*Sambucus nigra* L., also known as elder, elderberry, European elderberry, European lilac, and European elderberry, is a shrub commonly found in central and western Europe, as well as on other continents such as Africa, East Asia, New Zealand, and Australia [1]. Elderberry has long been known in traditional medicine and used, especially as both flower and fruit preparations (e.g., infusions or syrups) for the treatment of various diseases, especially viral infections of the respiratory system (cold and flu symptoms, influenza), fever, and as a diuretic agent [2,3,4]. Moreover, scientific research has proven its antiviral and anti-inflammatory effects [3].

Though flowers and fruits are known for their beneficial activity on human health, the knowledge of leaves of *S. nigra* is rather limited. Until now, the raw material has not been approved by international monographs and institutions (e.g., European Medicines Agency, European Scientific Cooperative on Phytotherapy, Food and Drug Administration). However, it is known that elderberry leaves are a rich source of polyphenols, predominantly flavonoids (mainly quercetin and kaempferol), and derivatives of these compounds (isoquercetin and rutin, as well as astragalin and myricetin, respectively), as well as of phenolic acids (chlorogenic acid, neochlorogenic acid, caffeic acid, p-coumaric acid, and dicavoylquinic acids) [2,5]. The elderberry leaves contain amino acids and tocopherols [6]. The literature data also posit that unripe berries of *S. nigra* may contain cyanogenic glycosides, of which the most abundant are sambunigrin and prunasin [7], which are non-toxic when intact, but endogenous plant enzymes may react with them and release hydrogen cyanide, causing potential toxic problems. However, it has been shown that cyanide degrades during heat treatment [2].

Scientific studies support the use of elderberry leaves in traditional medicine to treat skin inflammation, ulcers, burns, or boils and heal infected or chronic wounds [8]. Elderberry leaves have also been used to treat dermatological diseases (e.g., folliculitis and pemphigus) [9,10,11]. There are also reports in the literature on using ointments with the addition of elderberry ground leaves. An animal model study has also shown that adding 10% elderberry leaf extract to a cream used to treat third-degree burns promoted skin regeneration, causing healing and scarring [12]. Moreover, the literature data indicate that elderberry leaves possess antimicrobial, against, e.g., *Salmonella*’ strains, and antiparasitic activity [13]. Antiviral activity of the methanolic extract was detected against the dengue virus (DENV-2 serotype 2) [14]. In contrast, Daryani et al. described the in vitro antiprotozoal potential of methanolic preparations against *Toxoplasma gondii* tachyzoites [15]. Moreover, *S. nigra* leaves were also tested using different in vitro models, demonstrating their antioxidant properties [13,16]; however, their potential was often lower compared to the flowers’ or fruit extracts’ activity [5,13]. In a different investigation, the *S. nigra* leaf extracts were presented as anti-inflammatory [8] or anti-tyrosinase agents [17]. Both types of bioactivity, together with the antioxidant potential, are regarded as valuable in maintaining good skin condition.

Though the literature data provide some scant information on S. nigra leaves, data are lacking on natural raw material collected from different varieties of S. nigra. Therefore, to expand the knowledge on their phytochemical and healing potential, the aim of this study was the analysis of elderberry leaves of 12 varieties (“Sampo”, “Obelisk”, “Dwubarwny”, “Haschberg”, “Haschberg 1”, “Koralowy”, “Sambo”, “Black Beauty”, “Black Tower”, “Golden hybrid”, “Samyl”, “Samyl 1”) in two growth stages regarding their phytochemical characteristic and screening of their biological activity toward treating skin inflammatory diseases and wounds.

## 2. Results and Discussion

### 2.1. Phytochemical Analysis

Polyphenols are compounds with high biological activity [18]. Their presence in plants determines their therapeutic potential, particularly as antioxidants and anti-inflammatory agents [19]. The tested leaf extracts from 12 varieties of *S. nigra*, were chemically determined by estimating the sum of polyphenols and total flavonoid content with the colorimetric methods using the Folin–Ciocalteu and aluminum chloride reagents, respectively. Our results indicate a high and varied content of polyphenols in the tested extracts (Table 1). The total polyphenol content was the highest in extracts of “Sampo” leaves II (no. 2) (61.85 mg GAE/g), “Golden hybrid” leaves I (no. 19) (62.53 mg GAE/g), and “Golden hybrid” leaves II (no. 20) (60.60 mg GAE/g). The content of flavonoids in the tested extracts was also high. The highest quantity was detected in “Haschberg” leaves I and II extracts (no. 7 and no. 8) (10.35 mg QE/g and 10.79 mg QE/g, respectively), and “Golden hybrid” leaves I and II (no. 19 and no. 20) (10.40 mg QE/g, and 10.44 mg QE/g, respectively). Moreover, it was noticed that older leaves (leaves II) were usually characterized by a higher content of polyphenolic compounds but a lower total content of flavonoids compared to younger leaves (leaves I). The obtained results suggest that compounds from the polyphenol group constitute an essential part of the active compounds of the tested leaves from various varieties of *S. nigra*. The total content of polyphenols and the total content of flavonoids were examined for the first time for leaves of 12 different elderberry varieties at two stages of development. However, our data match the reports on the chemical composition of elderberry leaves. Nurzyńska-Wierdak et al. (2022) report that the total polyphenol content in water extracts from dry leaves of *S. nigra* was high and only slightly lower than in *S. nigra* flowers. According to its study, the dominant group of polyphenols were phenolic acids (2.32%) and flavonoids (1.48%) [20]. Skowrońska et al. (2022) determined that the total content of polyphenols in 70% ethanolic macerate and 70% ethanolic extract was 72.74 ± 6.89 μg GA/mg and 88.87 ± 11.21 μg GA/mg, which, similarly to our results, indicates a high content of polyphenols in the assessed raw material [8]. Skowrońska et al. (2022) also noticed that the macerate and water extract prepared in parallel contained fewer polyphenolic compounds than alcoholic preparations [8].

Furthermore, chlorogenic acid and quercetin content in all extracts was assessed (Table 1). An example chromatogram is shown in Figure S3 in the Supplementary Materials. The highest chlorogenic acid and quercetin contents were determined in extract “Golden hybrid” leaves I (no. 19). Previous literature data indicate the high efficiency of extraction of chlorogenic acid from *S. nigra*, where in the case of flowers, the best efficiency was obtained for high ethanol concentrations in the extraction mixture and a temperature of 50 °C [21]. It was noticed that chlorogenic acid content is higher in flowers than in leaves [22]. Notably, there are only a few reports regarding the content of active compounds in leaves, which justifies the need to address this topic. Additionally, this is the first attempt to assess the content of compounds in extracts from elderberry leaves of various varieties and stages of development.

### 2.2. Biological Activity

#### 2.2.1. Evaluation of Antioxidant Activity Using Cell-Free Methods

Since oxidative stress is a factor in the pathogenesis of many diseases [23], antioxidant activity is an important feature describing the therapeutic potential of plant extracts. Excess free radicals are pivotal in the pathogenesis of civilization diseases (e.g., diabetes, cancers, or cardiovascular diseases) [24]. Still, they may also reduce the ability of damaged tissues to regenerate, including delaying the wound-healing process [25]. Therefore, the characterization of their antioxidant potential is needed for new plants that have not yet been used in therapeutic approaches.

Therefore, we analyzed the ability to scavenge free radicals (DPPH^•^) by *S. nigra* varieties of leaf extracts. Moreover, we also tested the ability of extracts to reduce Cu(II) ions and chelate Cu(II) ions because, according to the available literature data, Cu(II) ions can cause cytotoxic and genotoxic effects on human cells, likely by enhancing the generation of reactive oxygen and nitrogen species [26]. The obtained results (Table 2) indicate that the extract from “Sampo” leaves I (no. 1) and leaves II (no. 2) have the highest free radical scavenging ability. The activity of extracts from the “Golden hybrid” variety was like that of the “Sampo” variety. The “Sampo” variety leaf extracts (no. 1 and no. 2) were also characterized by a high ability to reduce Cu(II) ions. Cu(II) ions were also strongly reduced by extracts from the leaves of the “Haschberg” variety (no. 7 and no. 8) and, similarly to the previous one, the “Golden hybrid” variety (no. 19 and no. 20). It was noticed that the extracts richest in polyphenolic compounds have the highest anti-free radical and Cu(II) ion-reducing ability. “Sampo” leaves I (no. 1) and “Golden hybrid” leaves I (no. 19) extracts, which were also characterized by the highest content of chlorogenic acid (Table 2), were particularly active in this area. The results of the Cu(II) ions’ chelating ability proved that the “Sampo” leaves I extract (no. 1) had the strongest activity. Moreover, Cu(II) ions were strongly chelated by the extract from the “Haschberg 1” leaves I (no. 9) and the “Koralowy” leaves I (no. 11). Our results indicate a weaker potential of extracts obtained from the older leaves to chelate Cu(II) ions.

While our research assessed for the first time the antioxidant potential of leaves collected from 12 various varieties of *S. nigra* in different stages of development, the literature data indicate that *S. nigra* leaves have free radical scavenging potential. Nurzyńska-Wierdak et al. (2022) suggested that the ability of aqueous extracts of *S. nigra* leaves to scavenge the DPPH radical is interesting (46.57%); however, it is lower than that of elderberry flowers and fruits [20]. A similar conclusion was obtained by Dawidowicz et al. (2006) examining ethanol–water (80:20 *v*/*v*) extract prepared using the ASE technique (Accelerated Solvent Extractor). Elderberry leaf extracts, especially those prepared at high temperatures (100 °C), scavenged the DPPH radical (48.52 ± 0.35%), but the activity was lower than that of flower or fruit extracts [5]. The recent study published by Skowrońska et al. (2022) reported that 70% ethanol macerate and extract from *S. nigra* leaves scavenged DPPH radical in a concentration-dependent manner and more intensely than aqueous products [8]. To the best of our knowledge, neither CUPRAC analysis nor Cu(II) chelating analysis to determine the antioxidant potential of elderberry leaves has been published; however, the FRAP analysis provided by Tundis et al. (2019), which measured the ability of ethanolic and methanolic extracts from leaves of *S. nigra,* indicated its ability to reduce the Fe(III) ions to Fe(II) [17].

#### 2.2.2. Evaluation of Enzyme Activity Inhibition Using Cell-Free Methods

Thanks to the content of various plant metabolites, plant extracts can modify the activity of multiple enzymes [27]. This feature, related to the increased activity of specific enzymatic proteins in many diseases, is a valuable parameter in the search for new drugs. Tyrosinase and hyaluronidase are essential enzymes that foster healthy human skin tissue. Tyrosinase is a copper-containing oxidase that plays a pivotal role in melanogenesis [28]; therefore, the high activity of this enzyme may lead to the accumulation of melanin in the skin and the appearance of unsightly discolorations [29]. Hyaluronidase degrades hyaluronic acid [30], and a lower concentration in the skin tissues can introduce dehydration and inflammation, probably associated with low molecular weight fragments of hyaluronic acid [31,32]. Moreover, hyaluronic acid is a natural biopolymer that plays a significant role in many biological processes, including wound healing [33]. As one of the most important components of the extracellular matrix, it facilitates the regeneration of damaged tissue, which is important in every phase of the healing process. Hyaluronan stimulates cellular migration, differentiation, and proliferation, and also participates in the regulation of extracellular matrix metabolism [33,34]. Thus, the degradation of hyaluronic acid under the influence of hyaluronidase may, therefore, delay the wound-healing process.

Our research showed that the analyzed extracts can inhibit these enzymes (Table 3). The inhibition of tyrosinase is 82.59 ± 1.03%–34.14 ± 4.69%; the hyaluronidase inhibition was estimated in the range of 41.28 ± 0.41–0.00%. Tyrosinase was inhibited most effectively by “Sambo” (no. 14), and “Golden hybrid” (no. 20) leaves II extracts, although all elderberry leaf extracts, except “Samyl” leaves II, had an enzyme-inhibition capacity of >25%. In turn, hyaluronidase was most strongly inhibited by “Sampo” leaves II extract (no. 2) and “Haschberg” leaves II (no. 8) (33.36 ± 2.51%). The “Sampo” leaves II extract (no. 2) showed the highest total ability to inhibit both enzymes (Table 3).

The data on *S. nigra* leaf extracts’ inhibitory potential on enzymes are scant. So far, Tundis et al. (2019) studied the dry alcoholic (methanol, ethanol) *S. nigra* leaf extracts and showed an anti-tyrosinase activity (IC_50_ = 204.5 ± 4.8 µg/mL and IC_50_ = 298.4 ± 3.3 µg/mL) [17]. Meanwhile, Mainka et al. (2021) have recently established the anti-hyaluronidase activity of water and ethanolic extracts from *S. nigra* leaves. However, the biological potential of extracts measured at a concentration of 500 µg/mL was lower than that found in our study (3.52 ± 2.94% and 2.92 ± 1.49%, respectively) [35]. In this study, we have shown that the extracts we tested contain chlorogenic acid. In the study by Zhou et al. (2023) [36], it was proven that chlorogenic acid showed a weak ability to inhibit hyaluronidase. This effect is in accordance with our results because we do not observe a significant increase in anti-hyaluronidase potential in regard to the content of chlorogenic acid in the extracts.

### 2.3. Principal Components Analysis

**Figure 1:**
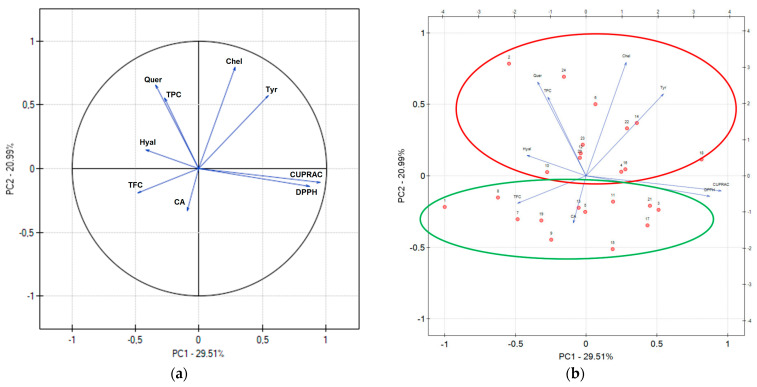

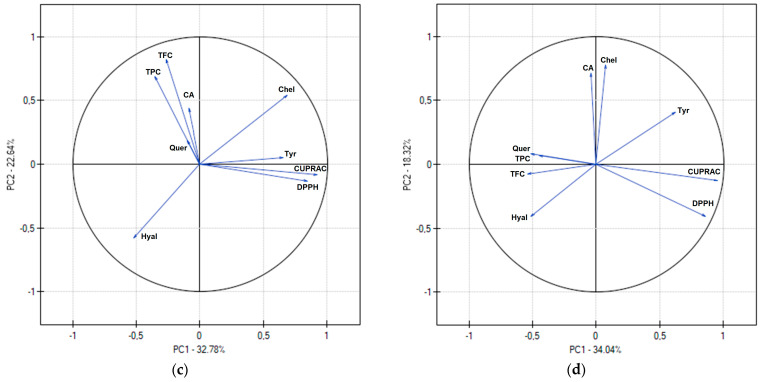
PCA analysis of all extracts (**a**), factor loading plot for all extracts (**b**), extracts from leaves I (**c**) and leaves II (**d**); where TPC—total phenolic content, TFC—total flavonoid content, CA—content of chlorogenic acid, Quer—content of quercetin, DPPH—antioxidant activity expressed in DPPH method, CUPRAC—antioxidant activity expressed in CUPRAC method, Chel—level of copper ion chelation, Tyr—percentage of inhibition of tyrosinase enzyme activity, Hyal—percentage of inhibition of hyaluronidase enzyme activity.

Principal components analysis (PCA) is a popular data analysis method that makes it possible to observe regularities between the studied variables. Figure 1 shows the results of the PCA analysis of extracts, considering all 24 extracts (Figure 1a), as well as those differentiated by the stage of development into leaves I (Figure 1c) and leaves II (Figure 1d).

In all cases, a negative correlation was observed between TPC and antioxidant activity, which indicates a significant contribution of polyphenolic compounds in this respect. A strong correlation was noticed between the antioxidant activity determined by the DPPH and CUPRAC methods, which indicates the equal contribution of different mechanisms of antioxidant action to the activity of the plant material. When analyzing the data for all extracts (Figure 1a), a positive correlation was demonstrated between the active components (chlorogenic acid and quercetin) content, which confirms that they are one of the most important polyphenolic compounds of the raw material, and total phenolic content (TPC), and a negative correlation between chlorogenic acid and the level of copper ion chelation. Figure 1b shows a clear division of all leaves I (green line) and leaves II (red line) extracts, which confirms some differences between plant materials at different development stages. Therefore, it is worth considering the dependencies in both groups. When analyzing a group of leaves I (Figure 1c), a correlation was noticed between the chlorogenic acid content and TPC, indicating that chlorogenic acid is one of the active compounds with the highest content in the plant material. Similarly, the correlation between quercetin and TFC indicates a significant compound content in the plant material. In the case of leaves II (Figure 1d), a positive correlation between chlorogenic acid and the level of copper ion chelation was visible. Regardless of the analytical group, a negative correlation was found between chlorogenic acid and quercetin levels for all results and for leaves I and II separately.

### 2.4. Evaluation of Wound-Healing Potential

**Figure 2:**
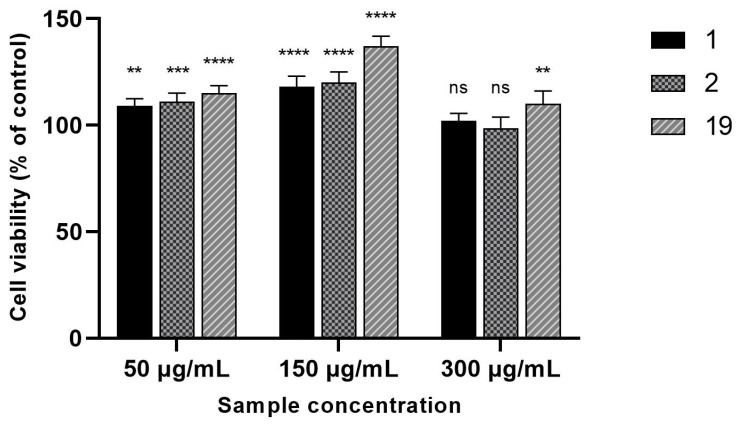
Viability of Hs27 cells incubated for 24 h with the increased concentrations of extracts 1 (“Sampo” leaves I), 2 (“Sampo” leaves II), and 19 (“Golden hybrid” leaves II). The results of MTT assay were statistically analyzed by ANOVA with a post hoc Dunnett’s test. Statistical significance (vs untreated Hs27 cells) was designated as **** when *p* < 0.0001; *** when *p* < 0.001; ** when *p* < 0.01. ns: not significant.

Evaluation of the wound-healing properties of extracts 1, 2, and 19 was preceded by the determination of their cytotoxic effect on normal human skin fibroblasts (Hs27 cells). None of the tested concentrations (i.e., 50, 150, 300 µg/mL) impaired the viability of Hs27 cells. On the contrary, the investigated extracts increased the proliferation of fibroblasts (Figure 2). The most beneficial effect was observed when the extracts were used at 150 µg/mL. At this concentration, the viability of Hs27 cells ranged from 118 to 137% (vs. untreated cells). Since fibroblast proliferation and migration capacity play pivotal roles in the wound-healing process, the extracts 1, 2, and 19 were examined in the scratch assay at the concentration that most strongly stimulated the proliferation of Hs27 cells (i.e., 150 µg/mL).

**Figure 3:**
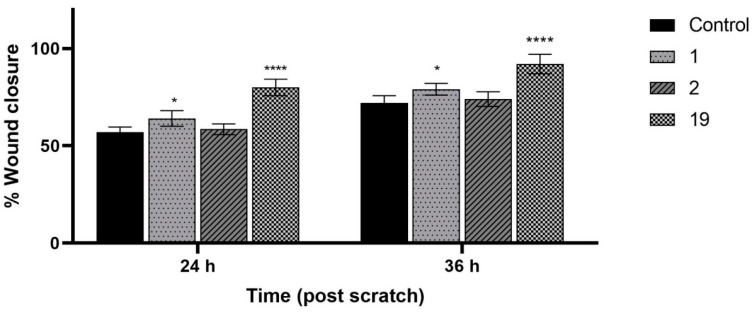
Wound-healing effect observed after 24 h and 36 h incubation of Hs27 cells with the investigated extracts 1 (“Sampo” leaves I), 2 (“Sampo” leaves II), and 19 (“Golden hybrid” leaves II). The samples were tested at the concentration of 150 µg/mL. Results were statistically analyzed by ANOVA with a post hoc Tukey’s test. Statistical significance was designated as “*” when *p* < 0.05 and “****” when *p* < 0.0001.

**Figure 4:**
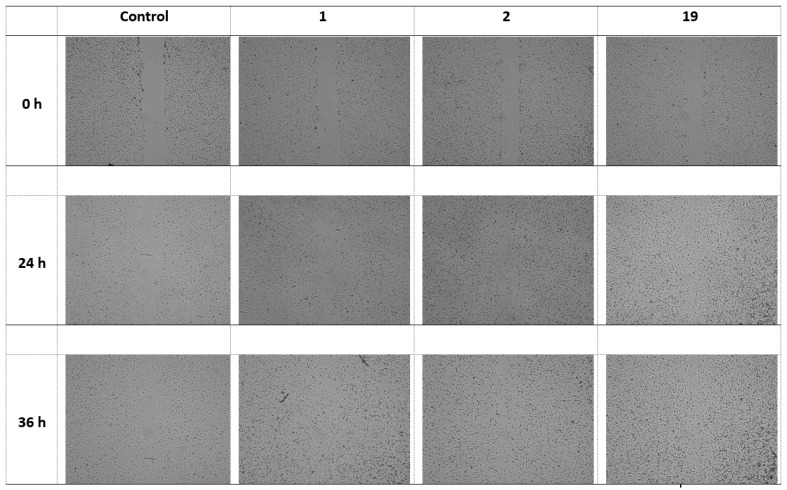
Graphical representation of wound-healing properties of 1 (“Sampo” leaves I), 2 (“Sampo” leaves II), and 19 (“Golden hybrid” leaves II), incubated for 24 and 36 h.

Then, the activity of extracts 1, 2, and 19 in wound treatment was assessed. The most active extract was 19 and closed the wound by 80.5 ± 4.1% (after 24 h) and 91.7 ± 5.0% (after 36 h), followed by extract 1, which closed the wound by 64.4 ± 3.7% (24 h) and 79.0 ± 3.0% (36 h). The activity of extract 2 did not differ statistically significantly from the control, which achieved 57.3 ± 3.1% (after 24 h) and 72.4 ± 4.2% (after 36 h) (Figure 3 and Figure 4).

Therefore, the results confirm the wound-healing potential of selected elderberry leaf species, demonstrated using human normal skin fibroblasts (Hs27 cells). Moreover, elderberry leaf extract was shown by Skowrońska et al. to have a supporting effect on wound healing and to modulate the inflammatory response by stimulating keratinocytes and the cellular release of TNF-α and interleukins -1β, -6, and -8 [37]. What is noteworthy is the high activity of the extract from the “Golden hybrid” variety (no. 19), which contains the highest amount of chlorogenic acid among all the tested extracts. Anti-inflammatory and promoting wound-healing properties of chlorogenic acid were previously demonstrated by other authors [38,39], who presented its valuable influence on the wound-healing process after systemic and topical application [40,41,42]. These data support our observation according to which this phenolic acid can have a regenerative effect on the skin when of the “Golden hybrid” variety.

## 3. Materials and Methods

### 3.1. Plant Material

The raw material for this study consisted of dried leaves of twelve varieties of elderberry (“Sampo”, “Obelisk”, “Dwubarwny”, “Haschberg”, “Haschberg 1”, “Koralowy”, “Sambo”, “Black Beauty”, “Black Tower”, “Golden hybrid”, “Samyl”, “Samyl 1”). The research tested 12 different elderberry genotypes entered into the National Register of Varieties. All varieties were collected from the Research Center on Cultivar Testing (COBORU) in Słupia Wielka, Greater Poland, Poland. The plant material for the analysis of its phytochemical composition came from the 12th year of the plantation run under the Post-Registration Variety Experimentation system. The leaves were collected at two stages of plant development, i.e., young leaves at the beginning of the vegetation period (leaves I; harvest time: 10 April 2023) and older leaves, i.e., during the flowering period (leaves II; harvest time: 15 May 2023). On each of the analyzed dates of sampling for analysis, this activity was performed in the morning so that the plant sample was taken at full turgor. In the agrotechnical category, the testing of 12 varieties of elderberry should be considered as an assessment of the biological progress of the tested species, providing objective information about the utility value of the tested varieties, as well as helping farmers make the right choice for growing the most valuable genotypes adapted to local farming conditions. Photos and description of leaves of individual varieties are included as Figure S6 and Table S1 in the Supplementary Materials.

### 3.2. Chemical Reagents

Chlorogenic acid (Phyproof^®^ Reference Substance) as well as quercetin (≥95%) were obtained from Sigma-Aldrich (Poznan, Poland). Sodium carbonate, sodium hydroxide, DMSO, formic acid, methanol, ammonium acetate, and copper (II) chloride were purchased from Avantor Performance Materials Poland S.A. (Gliwice, Poland). HPLC grade acetonitrile and water, as well as Folin–Ciocalteu phenol reagent, were from Merck (Darmstadt, Germany). Chlorogenic acid and gallic acid were purchased from Sigma-Aldrich (Poznań, Poland). All other chemicals were from Sigma-Aldrich Chemical Co. (Taufkirchen, Germany). High-quality pure water and ultra-high-quality pure water were prepared using a Direct-Q 3 UV Merck Millipore purification system (Burlington, MA, USA).

### 3.3. Extract Preparation

A total of 5 g of dried and crushed *S. nigra* leaves were weighed and placed in ground-glass conical flasks. Then, 50 mL of 70% methanol was poured in. The flasks were placed in an ultrasonic bath and extracted for 20 min at 50 °C. The process was carried out four times; each time, the extracts were filtered through cotton wool into larger flasks, and the plant material was poured with a new portion of solvent. The extracts were concentrated using a rota vapor to volume, i.e., of 25.0 mL to obtain the initial concentration of 0.2 g dry plant material/mL. The extracts were stored at −20 °C until the next analysis.

### 3.4. Determination of Phytochemical Profile

#### 3.4.1. Total Polyphenolic Content

Total polyphenolic content (TPC) was determined using the procedure described by Studzińska-Sroka et al. [43]. Briefly, a 96-well test plate was used for the study. A total of 25.0 μL of the tested extracts (at a concentration of 3.125 mg dry plant material/mL for all tested varieties) or individual dilutions of the gallic acid (0.526–0.016 mg/mL) were transferred to the wells. Then, 200.0 μL of distilled water, 15.0 μL of Folin–Ciocalteu reagent, and 60.0 μL of 20% aqueous sodium carbonate solution were added. In the blank test, the tested extract or gallic acid was replaced with the extraction solvent. The plates were shaken in the dark for 5 min at room temperature at 600 rpm. The next stage was a 25 min incubation in the dark at room temperature, without shaking. The absorbance was measured at a wavelength of λ = 760 nm using a plate reader (Multiskan GO 1510, Thermo Fisher Scientific, Vantaa, Finland). The experiment was repeated twice; the final value was the average of four results (*n* = 3). The total content of polyphenols in the tested extracts was calculated based on a calibration curve made for gallic acid and expressed as gallic acid equivalent [mg GAE/g dry plant material].

#### 3.4.2. Total Flavonoid Content

The total flavonoid content (TFC) was evaluated using the method described previously [44]. A total of 100.0 μL of the tested extract (at a concentration of 6.25 mg dry plant material/mL for all tested varieties) or individual dilutions of the quercetin (0.100–0.003 mg/mL) was transferred to a 96-well test plate. Then, 100.0 μL of 2% methanolic aluminum chloride solution was added. The tested extract or quercetin was replaced with the extraction solvent in the blank test. The plate was shaken at 300 rpm for 10 min at room temperature and in the dark. Using a plate reader (Multiskan GO 1510, Thermo Fisher Scientific, Vantaa, Finland), the absorbance measurement was taken at a wavelength of λ = 415 nm. The assay was repeated twice. The final result was the average of four (*n* = 6) measurements. The total content of flavonoids in the tested extracts was calculated based on the standard curve prepared for quercetin and expressed as quercetin equivalent [mg QE/g plant dry material].

#### 3.4.3. High-Performance Liquid Chromatography Analysis

The previously described HPLC method with UV detection was used to measure the content of chlorogenic acid and quercetin using Dionex Thermoline Fisher Scientific with Chromeleon software version 7.0. In brief, the LiChrospher RP-18 column (Merck, Darmstadt, Germany) with a 5 μm particle size and dimensions of 250 mm by 4 mm was used for the separations. Formic acid 0.1% (A) and acetonitrile (B) with gradient elution (0–10 min, 12% B; 10–60 min, 12–60% B; and 60–65 min, 12% B) were combined to create the mobile phase. The temperature of the column was kept at 30 °C, and the mobile phase flow rate was 0.6 mL/min. The substances were detected at a wavelength of 360 nm [45]. The presence of chlorogenic acid and quercetin in the extracts was confirmed by comparison of retention time and UV spectra of analyzed substances with their reference standards.

### 3.5. Antioxidant Activity

#### 3.5.1. DPPH Analysis

A previously described DPPH analysis was used to determine the antiradical activity [43]. A total of 25.0 μL of the tested extract (3.125–0.391 mg dry plant material/mL) and 175.0 μL of DPPH solution (0.2 mM in methanol) were transferred to a 96-well plate. The plates were shaken at 300 rpm in the dark for 30 min at room temperature. Using a plate reader (Multiskan GO 1510, Thermo Fisher Scientific, Vantaa, Finland), the absorbance measurement was taken at a wavelength of λ = 517 nm. The assay was repeated twice; the result was the average of four measurements (*n* = 4). The results are expressed as IC_50_ values ± SD. Trolox in the concentration range of 0.15–0.025 mg/mL was used as a positive control.

#### 3.5.2. CUPRAC Analysis

The CUPRAC analysis was conducted using the procedure described previously [43]. Solutions of neocuproine (7.5 mM), copper (II) chloride (10 mM), and ammonium acetate buffer (1 M, pH = 7.0) were mixed in a 1:1:1 *v*/*v* ratio to obtain the CUPRAC reagent. Then, 50 μL of the tested extract (1.563–0.195 mg/mL) and 150 μL of the CUPRAC reagent were mixed. The absorbance was measured after a 30 min incubation in a dark place and at room temperature, at a wavelength of λ = 450 nm. Blank was an attempt to replace 50.0 μL of the tested extract with 50.0 μL of extraction solvent. The assay was repeated twice. The result is the average of four determinations (*n* = 4). The results are expressed as IC_0.5_ values ± SD. Trolox in the 0.025–0.0016 mg/mL concentration range was used as a positive control.

#### 3.5.3. Chelating Cu^2+^ Analysis

The chelating potential was determined using the procedure described previously [46]. Briefly, 30.0 µL of the tested extract (12.5 mg/mL–1.563 mg/mL), 175.0 µL of buffer (50 mM, pH 6), and 30.0 µL of CuSO_4_ × 5H_2_O solution (0.4 mM) were mixed. The plate was incubated in the dark for 10 min at room temperature. In the next step, 15.0 µL of pyrocatechol violet (PV) solution (2 mM) was added to the wells and incubated again, shaking for 20 min at room temperature. The control was prepared without the examined sample; the blank was deprived of pyrocatechol violet. The absorbance measurement was taken at a wavelength of 632 nm. The assay was repeated twice; the result was the average of four measurements (*n* = 4). The results are expressed as IC_50_ values ± SD. Quercetin in the concentration range of 0.166–0.042 mg/mL was used as a positive control.

### 3.6. Enzyme Activity Inhibition

#### 3.6.1. Anty-Tyrosinase Activity

The experiment was performed on a 96-well plate. The determination was performed as follows. The anti-tyrosinase activity was measured using the method used previously by Studzińska-Sroka et al. [43]. Briefly, 75.0 µL of phosphate buffer (0.1 M; pH = 6.8), 50.0 µL of 192 U/mL tyrosinase solution, and 25.0 µL of the solution of the tested extract (100 mg/mL) were mixed in a 96-wells plate. The plate was shaken for 10 min at room temperature. In the next step, 50.0 µL of L-DOPA solution was added to each well and incubated for 20 min at 25 °C. The control was prepared without the sample but with the extraction solvent. The blank of the control contained extraction solvent instead of the sample and phosphate buffer, while the blank of the sample contained the buffer instead of the L-DOPA solution. The absorbance of the sample at wavelength was then measured λ = 475 nm. The assay was repeated twice. The final result was the average of four measurements (*n* = 3). The results are expressed as % of inhibition ± SD. Azelaic acid in the concentration range of 2.0–0.8 mg/mL was used as a positive control.

#### 3.6.2. Anty-Hyaluronidase Activity

The anti-hyaluronidase activity was performed according to the previously described method [43]. Briefly, 25.0 µL of incubation buffer, 25.0 µL of enzyme solution (30 U/mL), 10.0 µL of the tested extract (50 mg/mL), and 15.0 µL of acetate buffer were mixed in the well. After the 15 min. of incubation (37 °C) with shaking, 25.0 µL of hyaluronic acid (HA) solution was added and incubated for 45 min with shaking (37 °C). After this time, 200.0 µL of CTAB solution in 2% sodium hydroxide was added. After 10 min of incubation without shaking (at room temperature), the absorbance (λ = 600 nm) was measured using a plate reader (Multiskan GO 1510, Thermo Fisher Scientific, Vantaa, Finland). The composition of blanks was previously described [43]. The determination was repeated twice and calculated from four results (*n* = 4). The results are expressed as percentage of inhibition ± SD. β-Escin in the concentration range of 8.0–5.0 mg/mL was used as a positive control.

### 3.7. Cytotoxicity Assay

Using the MTT method, the vitality of human normal skin fibroblasts (Hs27 cells) cultured with extracts 1, 2, and 19 for 24 and 36 h was assessed using the methodology described in detail earlier [47].

### 3.8. Scratch Test

Using the scratch assay, the ability of extracts 1, 2, and 19 to heal wounds was investigated on Hs27 cells using the methodology described in detail earlier [47].

### 3.9. Statistical Analysis

The means ± SD were used to express the collected data. One-way analysis of variance (ANOVA) was used for statistical analysis, and Statistica 13.3 software (Statsoft, Krakow, Poland) was used to calculate statistical differences (using Duncan’s post hoc tests) with a significance threshold of *p* < 0.05. Principal component analysis (PCA) was used to analyze correlations using Statistica 13.1 and PQStat Software version 1.8.4.142. ANOVA statistically analyzed the results of MTT assay with a post hoc Dunnett’s test. Statistical significance (vs. untreated Hs27 cells) was designated as **** when *p* < 0.0001; *** when *p* < 0.001; ** when *p* < 0.01. ns—not significant. ANOVA was used to statistically analyze the results with a post hoc Tukey’s test. Statistical significance was designated as “*” when *p* < 0.05 and “****” when *p* < 0.0001.

## 4. Conclusions

In conclusion, our results indicate that extracts obtained from the leaves of 12 different varieties of *S. nigra* are characterized by attractive biological potential. Each of the tested extracts was characterized by antioxidant activity manifesting through various mechanisms of action. The variety that stood out in the antioxidant area was “Sampo” (leaves I). The extracts from the younger leaves were usually (without the “Obelisk” variety) more potent antioxidants than those from the older leaves. Regarding the influence of enzyme activity, the best multidirectional pro-health effect on the treatment of skin inflammation was specified for “Sampo” leaves II extract. Noteworthy, “Golden hybrid” leaves, rich in chlorogenic acid, were valuable plant material in wound treatment. The high TPC content in leaf extracts from all *S. nigra* varieties should not be neglected, and especially the high content of chlorogenic acid in the extracts (especially in a group of younger leaves) can be underlined. Moreover, based on statistical analysis, chlorogenic acid is probably one of the active ingredients that significantly influences the biological properties of the extracts. Interestingly, its effect differs in the case of extracts from younger and older leaves. The obtained results are encouraging for further studies of the most active varieties of *S. nigra*.

## Figures and Tables

**Table 1:** Total polyphenol content (TPC), total flavonoid content (TFC), and content of chlorogenic acid and quercetin in extracts.

	TPC		TFC		Chlorogenic Acid Content		Quercetin Content	
Extract No.	mg GAE/g	SD	mg QE/g	SD	mg/g	SD	mg/g	SD
1	44.69 ^e,f,g^	1.02	7.55 ^e,f,g^	0.39	17.55 ^j^	0.57	0.51 ^e^	0.05
2	61.85 ^a^	1.18	7.51 ^e,f,g^	0.43	16.76 ^k^	0.67	0.78 ^b^	0.08
3	41.74 ^g^	2.31	7.74 ^d,e,f^	0.64	20.22 ^g^	0.20	0.22 ^g^	0.03
4	46.13 ^e,f^	1.14	7.06 ^f,g,h^	0.50	12.75 ^ł^	0.27	0.26 ^g^	0.02
5	41.36 ^g^	1.31	9.05 ^b^	0.57	18.28 ^i,j^	0.28	0.60 ^d^	0.06
6	48.09 ^d,e^	0.98	6.74 ^h^	0.50	13.35 ^l,ł^	0.33	0.93 ^a^	0.03
7	52.32 ^b,c,d^	0.96	10.35 ^a^	0.64	27.49 ^b^	0.43	0.27 ^g^	0.02
8	54.94 ^b,c^	3.71	10.79 ^a^	0.59	19.52 ^g,h^	0.59	0.39 ^f^	0.03
9	51.42 ^c,d^	0.90	8.08 ^c,d,e^	0.42	26.93 ^b^	0.69	0.23 ^g^	0.02
10	56.14 ^b^	1.47	7.06 ^f,g,h^	0.41	23.07 ^e^	0.30	0.34 ^f^	0.03
11	46.13 ^e,f^	1.59	8.26 ^c,d^	0.34	13.89 ^l^	0.38	0.16 ^h^	0.02
12	51.24 ^c,d^	3.09	10.15	0.38	19.66 ^g,h^	0.66	0.69 ^c^	0.06
13	53.92 ^b,c^	0.91	7.07 ^g,h^	0.74	24.04 ^d^	0.40	0.36 ^f^	0.03
14	50.77 ^c,d^	2.65	5.67 ^i^	0.63	21.96 ^f^	0.69	0.37 ^f^	0.03
15	52.74 ^b,c^	1.57	6.05 ^i^	0.50	27.34 ^b^	0.37	0.10 ^h,i,j^	0.01
16	51.84 ^b,c,d^	2.55	5.66 ^i^	0.22	27.50 ^b^	0.57	0.12 ^h,i,j^	0.01
17	46.09 ^e,f^	3.48	6.79 ^h^	0.50	27.72 ^b^	0.27	0.15 ^h,i^	0.01
18	41.38 ^g^	1.52	5.52 ^i^	0.55	19.12 ^h^	0.29	0.14 ^h,i,j^	0.01
19	62.53 ^a^	2.56	10.40 ^a^	0.84	34.15 ^a^	0.24	0.08 ^j^	0.01
20	60.60 ^a^	4.27	10.44 ^a^	0.60	27.23 ^b^	0.27	0.09 ^i,j^	0.01
21	41.74 ^g^	1.67	8.02 ^c,d,e^	0.47	19.49 ^g,h^	0.39	0.25 ^g^	0.02
22	42.84 ^f,g^	0.91	8.52 ^b,c^	0.42	18.83 ^h,i^	0.38	0.27 ^g^	0.02
23	50.79 ^c,d^	4.75	8.34 ^c,d^	0.43	25.87 ^c^	0.58	0.81 ^b^	0.06
24	52.50 ^b,c^	2.25	7.20 ^f,g,h^	0.41	25.64 ^c^	0.46	0.90 ^a^	0.06

1—“Sampo” leaves I, 2—“Sampo” leaves II; 3—“Obelisk” leaves I, 4—“Obelisk” leaves II, 5—“Dwubarwny” leaves I, 6—“Dwubarwny” leaves II, 7—“Haschberg” leaves I, 8—“Haschberg” leaves II, 9—“Haschberg 1” leaves I, 10—“Haschberg 1” leaves II, 11—“Koralowy” leaves I, 12—“Koralowy” leaves II, 13—“Sambo” leaves I, 14—“Sambo” leaves II, 15—“Black Beauty” leaves I, 16—“Black Beauty” leaves II, 17—“Black Tower” leaves I, 18—“Black Tower” leaves II, 19—“Golden hybrid” leaves I, 20—“Golden hybrid” leaves II, 21—“Samyl” leaves I, 22—“Samyl” leaves II, 23—“Samyl 1” leaves I, 24—“Samyl 1” leaves II. Mean values within a column with the same letter are not significantly different at *p* = 0.05 using Duncan’s test. The results in the form of figures are placed in Supplementary Materials as Figures S1 and S2.

**Table 2:** Antioxidant activity measured using CUPRAC method, Cu(II) chelating method, and DPPH method.

	CUPRAC		Cu(II) Chelating		DPPH	
Extract No.	IC_0.5_ [mg/mL]	SD	IC_50_ [mg/mL]	SD	IC_50_ [mg/mL]	SD
1	0.35 ^a^	0.02	2.58 ^a^	0.28	0.94 ^a^	0.04
2	0.63 ^b^	0.03	4.82 ^h,i^	0.14	1.88 ^b,c^	0.04
3	1.21 ^n^	0.09	4.00 ^f,g^	0.17	3.05 ^j^	0.06
4	0.97 ^k^	0.03	3.88 ^e,f^	0.32	2.48 ^f,g^	0.06
5	0.89 ^h,i^	0.05	3.82 ^d,e,f^	0.27	2.68 ^g,h,i^	0.14
6	0.82 ^f^	0.03	4.77 ^h,i^	0.66	2.48 ^f,g^	0.13
7	0.69 ^c^	0.02	3.38 ^b,c,d^	0.34	2.05 ^c,d^	0.13
8	0.72 ^c,d^	0.02	3.94 ^e,f,g^	0.19	2.20 ^d,e^	0.08
9	0.83 ^f,g^	0.01	2.70 ^a^	0.34	2.47 ^f,g^	0.15
10	0.72 ^c,d^	0.02	3.75 ^c,d,e,f^	0.28	1.94 ^b,c^	0.11
11	0.91 ^i,j^	0.01	3.15 ^b^	0.24	2.47 ^f,g^	0.05
12	0.95 ^j,k^	0.06	4.35 ^g,h^	0.20	2.39 ^e,f^	0.10
13	0.84 ^f,g,h^	0.02	3.68 ^c,d,e,f^	0.31	2.09 ^c,d^	0.06
14	1.00 ^k,l^	0.01	3.84 ^d,e,f^	0.22	2.72 ^h,i^	0.22
15	0.95 ^j,k^	0.03	3.23 ^b,c^	0.25	2.55 ^f,g,h^	0.24
16	0.95 ^j,k^	0.03	4.88 ^i^	0.40	2.36 ^e,f^	0.27
17	1.08 ^m^	0.02	3.47 ^b,c,d,e^	0.32	2.83 ^j^	0.24
18	1.29 ^o^	0.04	4.56 ^h,i^	0.07	3.04 ^i^	0.08
19	0.76 ^d,e^	0.02	3.92 ^e,f,g^	0.47	1.82 ^b^	0.09
20	0.81 ^e,f^	0.02	4.46 ^h,i^	0.11	1.95 ^b,c^	0.04
21	1.21 ^n^	0.07	3.89 ^e,f,g^	0.28	3.06 ^j^	0.11
22	1.04 ^l,m^	0.02	5.36 ^j^	0.18	2.44 ^f^	0.17
23	0.88 ^g,h,i^	0.02	4.79 ^h,i^	0.19	2.19 ^d,e^	0.12
24	0.75 ^d^	0.01	6.44 ^k^	0.21	1.91 ^b,c^	0.12

1—“Sampo” leaves I, 2—“Sampo” leaves II; 3—“Obelisk” leaves I, 4—“Obelisk” leaves II, 5—“Dwubarwny” leaves I, 6—“Dwubarwny” leaves II, 7—“Haschberg” leaves I, 8—“Haschberg” leaves II, 9—“Haschberg 1” leaves I, 10—“Haschberg 1” leaves II, 11—“Koralowy” leaves I, 12—“Koralowy” leaves II, 13—“Sambo” leaves I, 14—“Sambo” leaves II, 15—“Black Beauty” leaves I, 16—“Black Beauty” leaves II, 17—“Black Tower” leaves I, 18—“Black Tower” leaves II, 19—“Golden hybrid” leaves I, 20—“Golden hybrid” leaves II, 21—“Samyl” leaves I, 22—“Samyl” leaves II, 23—“Samyl 1” leaves I, 24—“Samyl 1” leaves II. Mean values within a column with the same letter are not significantly different at *p* = 0.05 using Duncan’s test. The results (extracts and positive controls) can be found in Figure S4 of the Supplementary Materials.

**Table 3:** Tyrosinase and hyaluronidase inhibition.

	Tyrosinase Inhibition		Hyaluronidase Inhibition	
Extract No.	[%]	SD	[%]	SD
1	34.9 ^k^	2.77	19.27 ^c^	1.76
2	65.22 ^b^	4.13	41.28 ^a^	0.41
3	56.71 ^c,d,e^	3.07	15.11 ^c,d^	3.67
4	63.71 ^b,c^	3.13	11.95 ^d,e,f,g^	0.95
5	35.82 ^k^	2.96	2.31 ^h^	0.34
6	61.56 ^b,c,d^	6.51	7.69 ^g^	0.96
7	40.45 ^i,j,k^	3.33	12.59 ^d,e,f^	5.76
8	38.77 ^j,k^	5.93	33.36 ^b^	2.51
9	34.14 ^k^	4.69	18.29 ^c^	3.40
10	48.20 ^f,g,h^	5.08	8.14 ^f,g^	5.24
11	65.41 ^b^	3.23	3.28 ^h^	4.32
12	55.09 ^d,e,f^	4.09	1.57 ^h^	0.46
13	46.94 ^g,h,i^	2.4	3.17 ^h^	1.41
14	80.33 ^a^	0.57	9.73 ^e,f,g^	3.24
15	43.54 ^h,i,j^	3.33	10.07 ^e,f,g^	3.08
16	66.45 ^b^	6.06	18.53 ^c^	2.92
17	65.06 ^b^	1.96	13.44 ^d,e^	3.61
18	75.98 ^a^	2.04	11.41 ^d,e,f,g^	3.77
19	53.51 ^e,f,g^	3.24	2.47 ^h^	1.10
20	82.59 ^a^	1.03	3.09 ^h^	1.87
21	44.43 ^h,i,j^	4.23	0.00 ^h^	0.00
22	66.07 ^b^	3.62	0.00 ^h^	0.00
23	59.07 ^b,c,d,e^	7.77	1.88 ^h^	0.72
24	59.23 ^b,c,d,e^	3.01	8.00 ^f,g^	2.07

1—“Sampo” leaves I, 2—“Sampo” leaves II; 3—“Obelisk” leaves I, 4—“Obelisk” leaves II, 5—“Dwubarwny” leaves I, 6—“Dwubarwny” leaves II, 7—“Haschberg” leaves I, 8—“Haschberg” leaves II, 9—“Haschberg 1” leaves I, 10—“Haschberg 1” leaves II, 11—“Koralowy” leaves I, 12—“Koralowy” leaves II, 13—“Sambo” leaves I, 14—“Sambo” leaves II, 15—“Black Beauty” leaves I, 16—“Black Beauty” leaves II, 17—“Black Tower” leaves I, 18—“Black Tower” leaves II, 19—“Golden hybrid” leaves I, 20—“Golden hybrid” leaves II, 21—“Samyl” leaves I, 22—“Samyl” leaves II, 23—“Samyl 1” leaves I, 24—“Samyl 1” leaves II. Mean values within a column with the same letter are not significantly different at *p* = 0.05 using Duncan’s test. The results (extracts and positive controls) can be found in Figure S5 of the Supplementary Materials.

## Data Availability

All data supporting reported results can be found within the manuscript and Supplementary Materials.

## Supplementary Materials

The following supporting information can be downloaded at: https://www.mdpi.com/article/10.3390/ph17050618/s1, Figure S1: Total polyphenol content (TPC) and total flavonoid content (TFC) in extracts; numbers 1–24 indicate the extracts prepared from different varieties of *S. nigra*; Figure S2: Content of chlorogenic acid and quercetin in extracts; numbers 1–24 indicate the extracts prepared from different varieties of *S. nigra*; Figure S3: Chromatogram of “Sampo” leaves I extract in the initial concentration; Figure S4: Antioxidant activity measured using DPPH method, CUPRAC method, Cu(II) chelating method; numbers 1–24 indicate the extracts prepared from different varieties of *S. nigra*; Figure S5: Inhibition of enzymes: tyrosinase (100 mg/mL) and hyaluronidase (50 mg/mL); numbers 1–24 indicate the extracts prepared from different varieties of *S. nigra*; Figure S6: Photos of leaves of individual varieties; Table S1: Characteristic features of elderberry varieties leaves.
