# Supplementary material for: Elderberry Leaves with Antioxidant and Anti-Inflammatory Properties as a Valuable Plant Material for Wound Healing
Source: Pharmaceuticals (Basel). 2024 May 10;17(5):618. doi: 10.3390/ph17050618 (PMC11124386; doi:10.3390/ph17050618)
Supplement: Supplementary file 1 [file pharmaceuticals-17-00618-s001.zip › pharmaceuticals-2967502-supplementary.pdf]

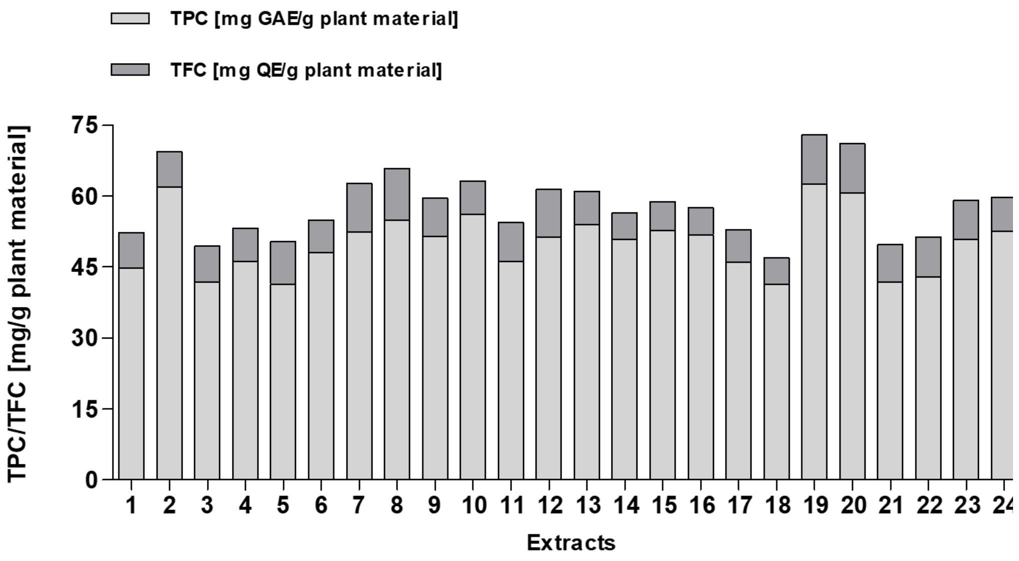

Figure S1. Total polyphenol content (TPC) and total flavonoid content (TFC) in extracts; numbers 1-24 indicate the extracts prepared from different varieties of *S. nigra*: 1-'Sampo' leaves I, 2-'Sampo' leaves II; 3-'Obelisk' leaves I, 4-'Obelisk' leaves II, 5-'Dwubarwny' leaves I, 6-'Dwubarwny' leaves II, 7-'Haschberg' leaves I, 8- 'Haschberg' leaves II, 9-''Haschberg 1' leaves I, 10- 'Haschberg 1' leaves II, 11-'Koralowy' leaves I, 12-'Koralowy' leaves II, 13-'Sambo' leaves I, 14-'Sambo' leaves II, 15-'Black Beauty' leaves I, 16-'Black Beauty' leaves II, 17-'Black Tower' leaves I, 18-'Black Tower' leaves II, 19-'Golden hybrid' leaves I, 20-'Golden hybrid' leaves II, 21-'Samyl' leaves I, 22-'Samyl' leaves II, 23-'Samyl 1' leaves I, 24-'Samyl 1' leaves II.

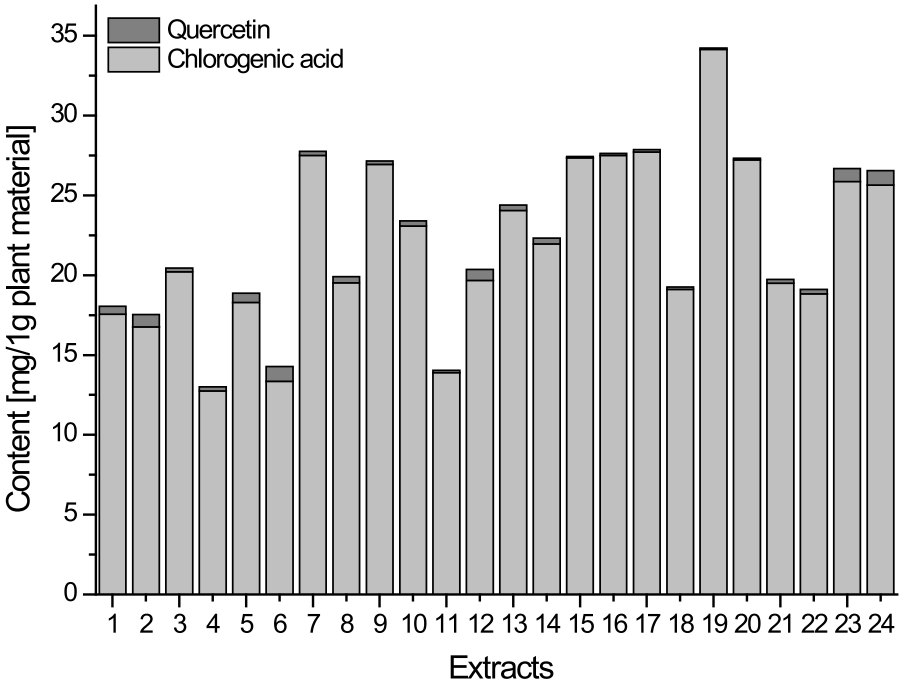

Figure S2. Content of chlorogenic acid and quercetin in extracts; numbers 1-24 indicate the extracts prepared from different varieties of *S. nigra*: 1-'Sampo' leaves I, 2-'Sampo' leaves II; 3-'Obelisk' leaves I, 4-'Obelisk' leaves II, 5-'Dwubarwny' leaves I, 6-'Dwubarwny' leaves II, 7-'Haschberg' leaves I, 8- 'Haschberg' leaves II, 9-''Haschberg 1' leaves I, 10- 'Haschberg 1' leaves II, 11-'Koralowy' leaves I, 12-'Koralowy' leaves II, 13-'Sambo' leaves I, 14-'Sambo' leaves II, 15-'Black Beauty' leaves I, 16-'Black Beauty' leaves II, 17-'Black Tower' leaves I, 18-'Black Tower' leaves II, 19-'Golden hy-brid' leaves I, 20-'Golden hybrid' leaves II, 21-'Samyl' leaves I, 22-'Samyl' leaves II, 23-'Samyl 1' leaves I, 24-'Samyl 1' leaves II.

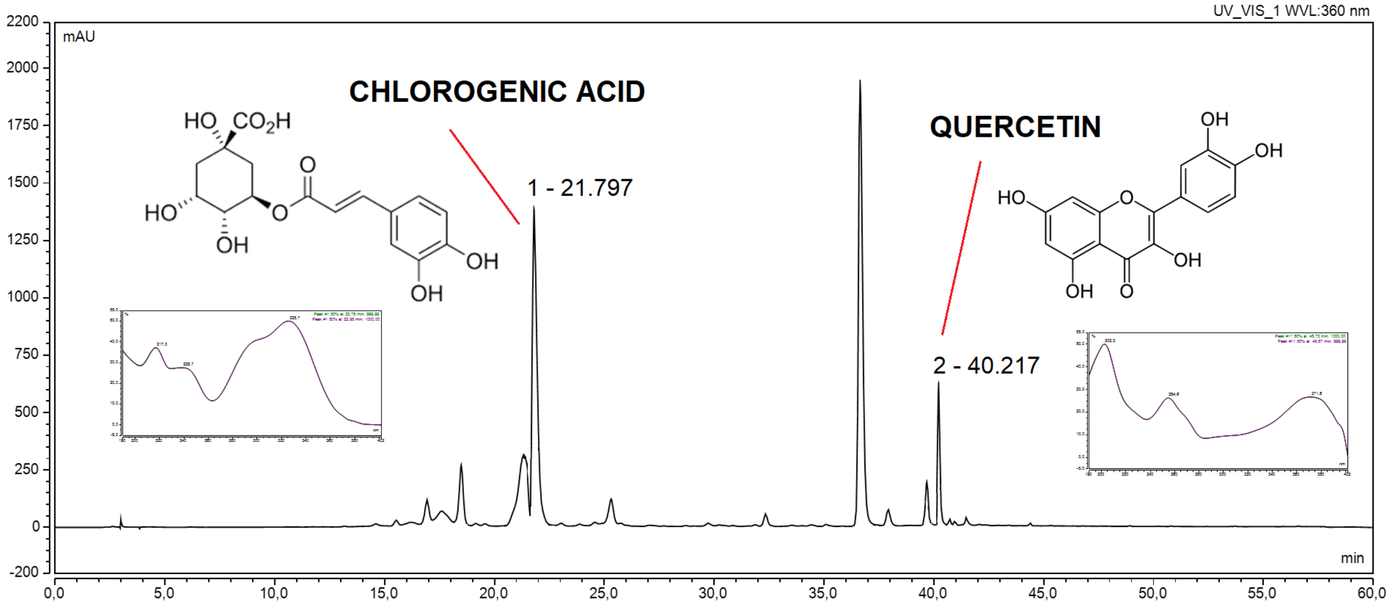

Figure S3. Chromatogram of Sampo leaves I extract in the initial concentration.

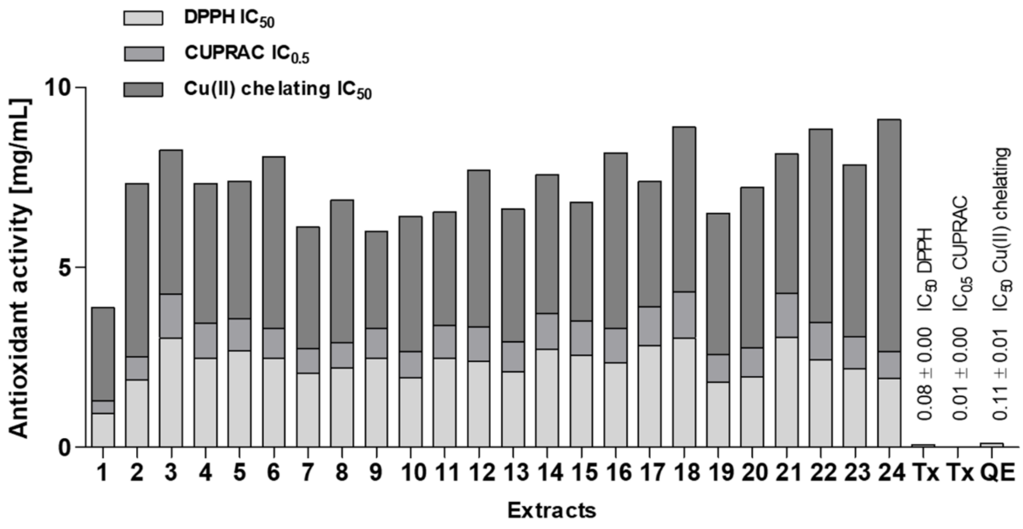

Figure S4. Antioxidant activity measured using DPPH method, CUPRAC method, Cu(II) chelating method; numbers 1-24 indicate the extracts prepared from different varieties of *S. nigra*: 1-'Sampo' leaves I, 2-'Sampo' leaves II; 3-'Obelisk' leaves I, 4-'Obelisk' leaves II, 5-'Dwubarwny' leaves I, 6-'Dwubarwny' leaves II, 7-'Haschberg' leaves I, 8- 'Haschberg' leaves II, 9-''Haschberg 1' leaves I, 10- 'Haschberg 1' leaves II, 11-'Koralowy' leaves I, 12-'Koralowy' leaves II, 13-'Sambo' leaves I, 14-'Sambo' leaves II, 15-'Black Beauty' leaves I, 16-'Black Beauty' leaves II, 17-'Black Tower' leaves I, 18-'Black Tower' leaves II, 19-'Golden hybrid' leaves I, 20-'Golden hybrid' leaves II, 21-'Samyl' leaves I, 22-'Samyl' leaves II, 23-'Samyl 1' leaves I, 24-'Samyl 1' leaves II; Tx-Trolox, QE-quercetin.

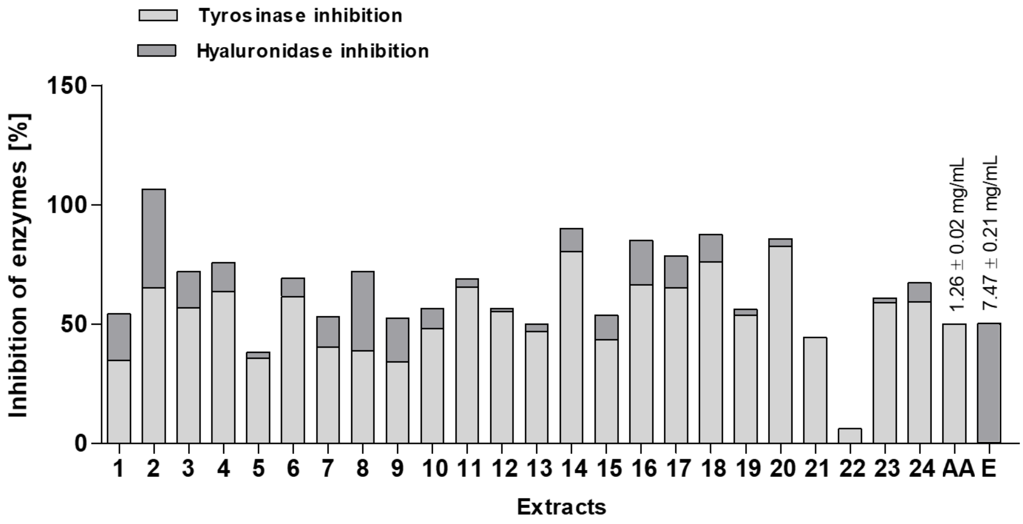

Figure S5. Inhibition of enzymes: tyrosinase (100 mg/mL) and hyaluronidase (50 mg/mL); numbers 1-24 indicate the extracts prepared from different varieties of *S. nigra*: 1-'Sampo' leaves I, 2-'Sampo' leaves II; 3-'Obelisk' leaves I, 4-'Obelisk' leaves II, 5-'Dwubarwny' leaves I, 6-'Dwubarwny' leaves II, 7-'Haschberg' leaves I, 8- 'Haschberg' leaves II, 9-''Haschberg 1' leaves I, 10- 'Haschberg 1' leaves II, 11-'Koralowy' leaves I, 12-'Koralowy' leaves II, 13-'Sambo' leaves I, 14-'Sambo' leaves II, 15-'Black Beauty' leaves I, 16-'Black Beauty' leaves II, 17-'Black Tower' leaves I, 18-'Black Tower' leaves II, 19-'Golden hybrid' leaves I, 20-'Golden hybrid' leaves II, 21-'Samyl' leaves I, 22-'Samyl' leaves II, 23-'Samyl 1' leaves I, 24-'Samyl 1' leaves II; AA-Azelaic acid, E-β-escin.

'Sampo'

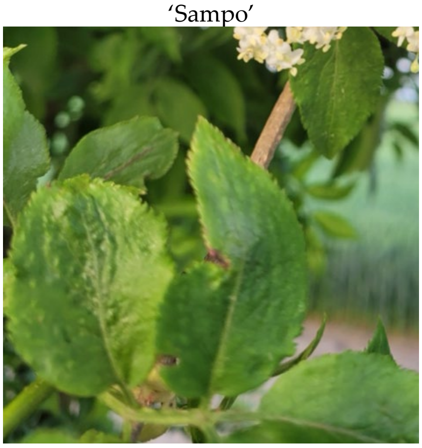

'Obelisk'

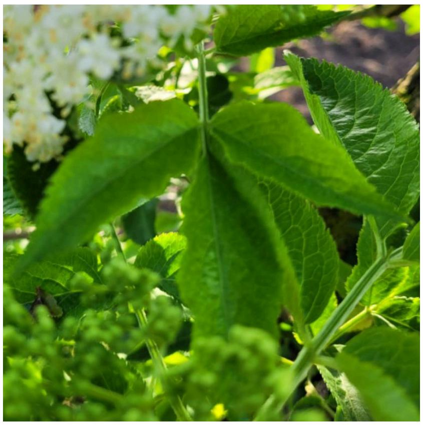

'Dwubarwny'

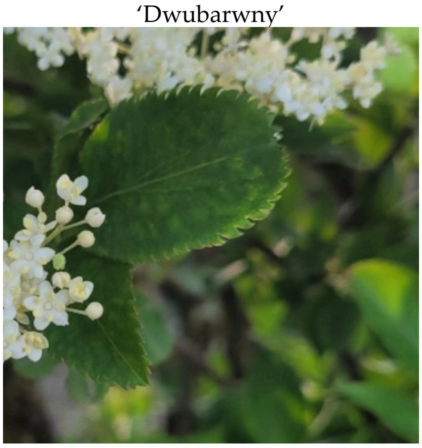

'Haschberg'

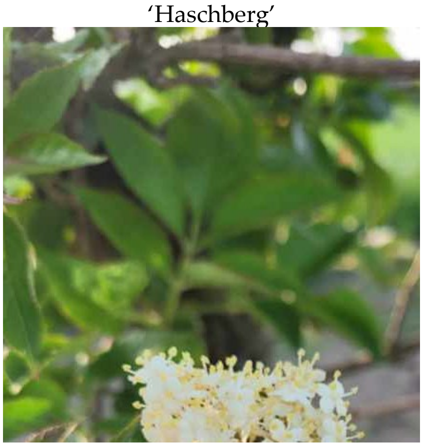

'Haschberg 1'

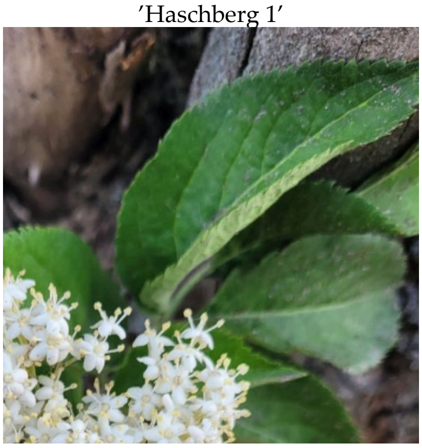

'Koralowy'

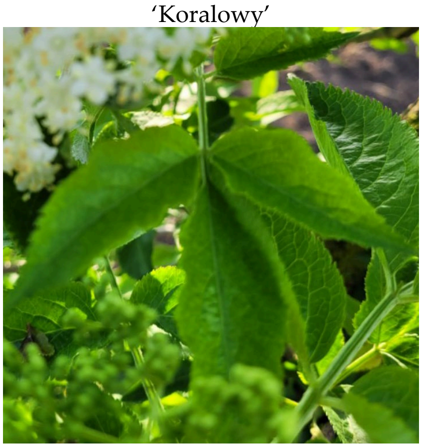

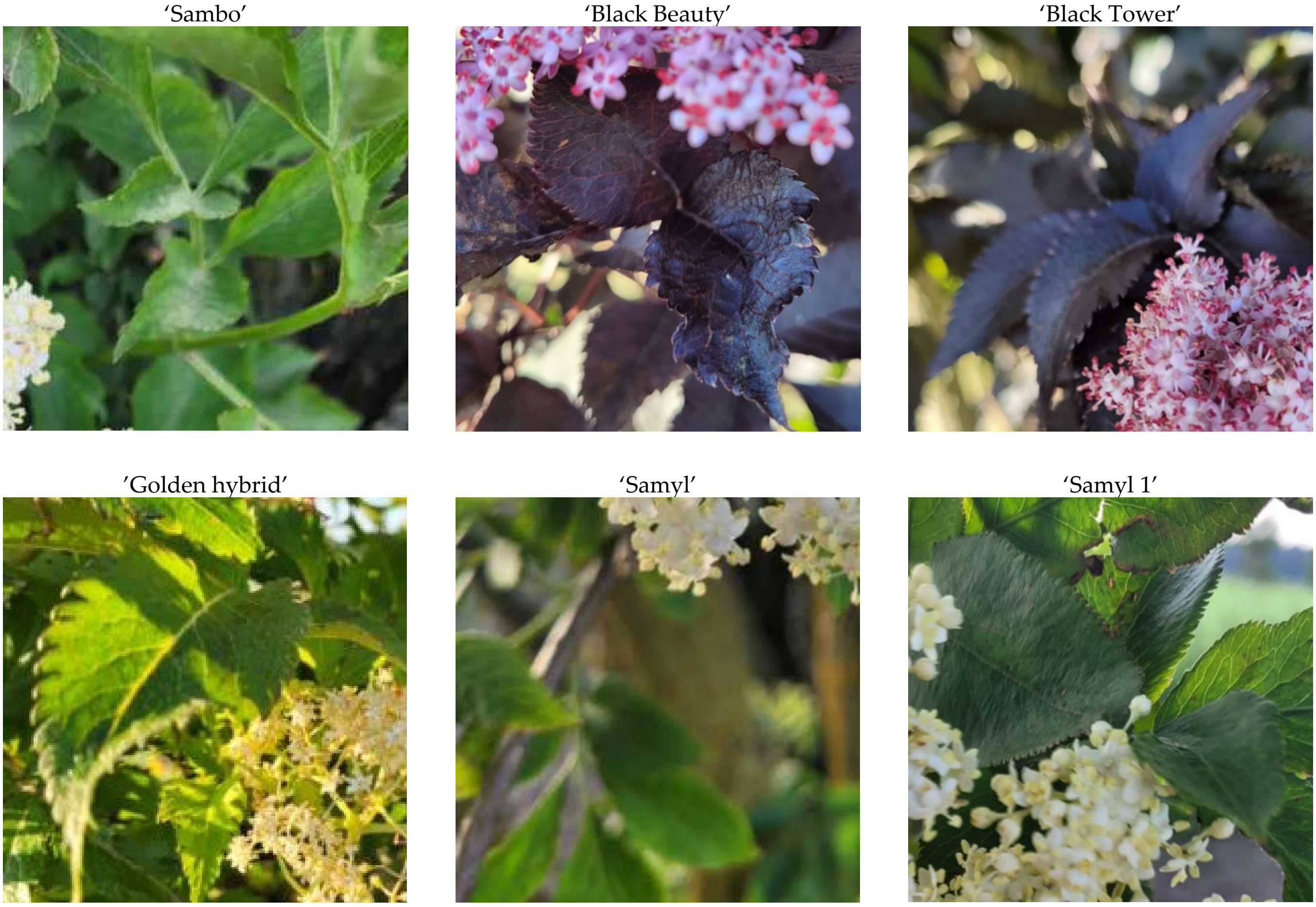

Figure S6. Photos of leaves of individual varieties

Table S1. Characteristic features of elderberry varieties leaves

Plant part	Yong Leaf	Leaf		Leaflet				
Variety / Characteristic features	Anthocyanin coloration*	Anthocyanin coloration	Relative position of leaflets	Lenght	Width	Undulation of margin	Glossiness on upper side	Length of petiolule
'Sampo'	weak	absent	touching	short	narrow	very weak to weak	weak	short to medium
'Obelisk'	absent or very weak	absent	free	medium to long	medium to broad	weak	weak	short to medium
'Dwubarwny'	absent or very weak	absent	free	short	narrow	weak	weak	medium
'Haschberg'	absent or very weak	absent	overlapping	long	broad	weak to medium	weak	Medium to long
'Haschberg 1'	absent or very weak	absent	overlapping	medium to long	broad	medium	medium	medium
'Koralowy'	absent or very weak	absent	touching	short	narrow to medium	medium	weak	very short
'Sambo'	weak	absent	touching	medium	medium	weak	weak to medium	short to medium
'Black beauty'	very strong	strong	free	long	narrow	medium	strong	very short
'Black tower'	medium	strong	free	short	very narrow to narrow	weak to medium	medium	very short
'Golden hybrid'	medium	weak	touching	medium	medium to broad	medium	medium	medium

**'Samyl'**	absent or very weak	absent	touching	short	narrow to medium	weak	weak to medium	short to medium
**'Samyl 1'**	absent or very weak	absent	touching	short	narrow	weak	weak	short to medium

*Anthocyanin coloration during rapid growth
